# Molecular Basis of N,N-Diethyl-3-Methylbenzamide (DEET) in Repelling the Common Bed Bug, *Cimex lectularius*

**DOI:** 10.3389/fphys.2017.00418

**Published:** 2017-06-20

**Authors:** Feng Liu, Xiaoming Xia, Nannan Liu

**Affiliations:** Department of Entomology and Plant Pathology, Auburn UniversityAuburn, AL, United States

**Keywords:** bed bug, DEET, olfactory receptor neuron, odorant receptor, sensilla

## Abstract

As the most extensively used chemical repellent, N,N-diethyl-3-methylbenzamide (DEET) displayed repellency to a wide range of insects, including the common bed bug, *Cimex lectularius*. While the neuronal or molecular basis involved in DEET's repellency have been majorly focused on mosquitos and fruit flies, DEET's repellency to the common bed bug is largely unreached. To gain new insights into the cellular and molecular mechanisms in DEET's repellency to the common bed bug, we characterized the neuronal response of bed bugs to DEET, identified the olfactory receptors targeted by DEET and demonstrated the interfering effect of DEET on bed bug's responses to human odorants. High doses of DEET were required for activating the olfactory receptor neurons in the sensilla of bed bugs and at least three DEET-sensitive receptors were functionally deciphered. These DEET-sensitive receptors presented even more sensitive to certain botanical terpenes/terpenoids which also displayed repellency at varying levels for bed bugs. In addition, DEET produced a blocking effect on the neuronal responses of bed bugs to specific human odors and showed inhibitory effect on the function of odorant receptors in responding to certain human odors. Taken together, our results indicate that DEET may function as a stimulus that triggers avoidance behaviors and a molecular “confusant” for interrupting the host odor recognition in the odorant receptors of bed bugs. The receptors that coincidently responded to both synthetic DEET and botanical terpenes/terpenoids suggested that DEET probably target on receptors that originally responded to terpenes/terpenoids. This study gave novel insight into the mechanisms of DEET's repellency to bed bugs and also provided valuable information for developing new reagents for bed bug control.

## Introduction

As an ectoparasite and obligate blood-feeding insect, bed bugs rely heavily on human and animal blood sources for survival, development, and reproduction. Compared to other blood-feeding arthropods (e.g., black flies, mosquitoes, body lice, fleas, and ticks), which also serve as disease vectors, bed bugs have long been considered to lack the capacity for disease transmission (Silverman et al., 2001). However, this may not be entirely correct, as a new study has indicated that bed bugs may transmit the protozoan *Trypanosoma cruzi*, which causes Chagas disease (Salazar et al., 2015). Nevertheless, the biting nuisance from a bed bug infestation still presents a huge stress and disturbance to human hosts, both physically and psychologically. To efficiently control this pest, insecticides, especially DDT and pyrethroids (Ter Poorten and Prose, 2005; Gangloff-Kaufmann et al., 2006), have been extensively used to suppress bed bug populations worldwide and in many developed countries or regions bed bugs were considered to be efficiently controlled and out of public concern. However, at the end of the 1990s, bed bugs showed a resurgent trend in several developed countries (Ter Poorten and Prose, 2005; Wang et al., 2013), partly as a result of the banning of highly efficient insecticides and the development of insecticide resistance (Romero et al., 2007; Yoon et al., 2008; Zhu et al., 2013).

As one of the most successful synthetic chemical repellents, DEET played and is still playing a critical role in insect management. DEET displays repellency to a wide range of insect species, including fruit flies (*Drosophila melanogaster*), mosquitos (*Aedes aegypti*; *Anopheles gambiae*; *Culex quinquefacistus*), kissing bug (*Triatoma rubida*), the common bed bug (*Cimex lectularius*), and the tropical bed bug (*Cimex hemipterus*) (Kumar et al., 1995; Badolo et al., 2004; Syed and Leal, 2008; Syed et al., 2011; Terriquez et al., 2013; Wang et al., 2013). As indicated by Wang et al. (2013), 10% of DEET repelled more than 94% of the common bed bug for 9 h with the presence of a host cue, carbon dioxide and 25% of DEET showed high repellency to bed bugs in a 14-day period. For the tropical bed bugs, which is a very close relative of the common bed bug, Kumar et al. (1995) also found that 75% of DEET showed 85% of repellency after 2 h treatment and 42% of repellency after 6 h treatment on the skin of rabbits.

Currently, two mechanisms are being proposed in the literature for the spatial repellency of DEET to the insects. The first is that DEET can act as a “confusant,” interfering in odorant recognition within the insect olfactory receptor neurons (ORNs) or odorant receptors (ORs) (Ditzen et al., 2008; Bohbot et al., 2011; Pellegrino et al., 2011; Bohbot and Dickens, 2012); the other one is that DEET acts as “stimulus” in repelling insects by activating the ORNs or ORs, resulting in the avoidance behavior (Syed and Leal, 2008; Xu et al., 2014). For example, Ditzen et al. (2008) found that DEET could significantly block the neuronal response of *An. gambiae* to one human odorant, 1-octen-3-ol, while another study by Pellegrino et al. (2011) indicated that DEET somehow scrambled the olfactory responses of *D. melanogaster* to odors and Bohbot et al. (2011) reported that DEET significantly inhibited the function of mosquitos' odorant receptors in response to the odorants. All these studies suggest interfering effect of DEET on the function of the insect olfactory system. Simultaneously, several other studies actually identified specific ORNs or olfactory receptors (e.g., OR136b in mosquito *Cx. quinquefaciatus*) that were activated by DEET, with marked electrophysiological responses (Syed and Leal, 2008; Xu et al., 2014), suggesting the activating effect of DEET on the insect olfactory system.

With all these studies have been focused on either fruit fly or mosquitoes, rare knowledge involved in the role of DEET in repelling the common bed bug has been revealed. Therefore, in this study we sought to reveal the possible mechanisms involved in the repellency of DEET to the common bed bug by testing their olfactory neuronal responses to DEET, identifying their DEET-sensitive odorant receptors and also investigating the interfering effect of DEET on bed bugs' responses to certain host cues from human odorants.

## Materials and methods

### Insects and compounds

The *C. lectularius* colony utilized in this study was originated from Ft. Dix, New Jersey, USA. This strain is susceptible to pyrethroid insecticides (Romero et al., 2007). All the common bed bugs were reared at 25 ± 2°C under a photoperiod of 12:12 (L: D). DEET and compounds were purchased from Sigma-Aldrich at high purity and diluted (v/v for liquid and w/v for solid) in dimethyl sulfoxide (DMSO) as indicated. Chemical Abstracts Service (CAS) numbers and purity are as follows: DMSO (67-68-5, 100%); pentanal (110-62-3, 97%); 2-butanone (78-93-3, 99.7%); 2-heptanone (107-87-9, 99%); 2-hexanone (591-78-6, 96%); hexanal (66-25-1, 98%); heptanal (111-71-7, 92%); octenal (124-13-0, 99%); nonanal (124-19-6, 95%); decanal (112-31-2, 98%); toluene (108-88-3, 99.8%); xylene (106-42-3, 99.5%); styrene (100-42-5, 99%); propylbenzene (103-65-1, 98%); ethylbenzene (100-41-4, 99%); 2,4-dimethylhexane (589-43-5, 99%); propylamine (107-10-8, 99%); butylamine (109-73-9, 99.5%); DEET (134-62-3, 97%); eugenol (97-53-0, 99%); carvacrol (499-75-2, 98%); linalyl acetate (115-95-7, 97%); menthyl acetate (89-48-5,97%); (−)-linalool (126-91-0, 95%); (+)-menthone (3391-87-5, 98.5%); citral (5392-40-5, 96%); geranyl acetate (105-87-3, 98%); 1S-(+)-3-carene (498-15-7, 99%); (+)-β-pinene (19902-08-0, 95%); α-terpineol (10482-56-1, 96%).

### Single sensillum recordings

Single sensillum recordings were performed as described previously (Liu et al., 2014; Liu and Liu, 2015). Female adult bed bug were selected for experiment at least 5 days after blood feeding.

To test the responses to DEET, 10 μl desired concentration of DEET was applied onto a filter paper strip (3 × 40 mm) and inserted into a Pasteur pipette to create the stimulus cartridge. While in investigating the potential influence of DEET on bed bug's response to odorants, we followed the method of Pellegrino et al. (2011) by pipetting undiluted DEET (10 μl) onto a filter paper strip and the desired concentration of odorants (10 μl) onto a second filter paper strip (Pellegrino et al., 2011). Both filter paper strips were then carefully inserted into a glass Pasteur pipette. A constant airflow across the antennae was maintained at 20 ml/s throughout the experiment. Humidified air was delivered to the antenna through a glass tube with a small hole, about 10 cm from the end of the tube. Stimulation was achieved by inserting the tip of the stimulus cartridge into the hole on the glass tube. A stimulus controller (Syntech, Germany) diverted a portion of the air stream (0.5 l/min) to flow through the stimulus cartridge for 0.5 s, thus delivering the stimulus to the sensilla. At least six replicates for each recording experiment with different stimuli were conducted on different individuals. As a high number of ORNs are co-located in each sensillum type, we did not attempt to calculate the firing rate for each ORN within the same sensillum. Instead, the total numbers of action potentials were counted off-line in a 500 ms period before and after stimulation for the whole sensillum. The number of action potentials after stimulation was subtracted from the number of action potentials before stimulation and multiplied by two in order to quantify the firing rate change in one sensillum in spikes per second.

### Expression of bed bug odorant receptors (ORs) in *Xenopus* oocyte system and two-electrode, voltage-clamp electrophysiological recordings

Bed bug ORs and ORCO (odorant receptor co-receptor) were amplified as previous descried (Liu et al., 2017). Basciallly, the ORs and ORCO were cloned into pT7Ts vector (a gift from Dr. Wang, Institute of Plant Protection in Chinese Academy of Agricultural Science, Beijing, China). The constructed vectors were linearized with specific restriction enzyme and cRNAs were synthesized from linearized vectors with mMESSAGE mMACHINE T7 (Ambion, Carlsbad, CA).

Mature healthy oocytes (stage V–VII) (Nasco, Salida, CA) were harvested from the African Clawed Frog (*Xenopus laevis*) and treated with collagenase I (GIBCO, Carlsbad, CA) in washing buffer [96 mM NaCl, 2 mM KCl, 5 mM MgCl_2_, and 5 mM HEPES (pH = 7.6)] for about 1 h at room temperature. After being cultured overnight at 18°C, oocytes were microinjected with 10 ng cRNAs of both OR and ORCO. After injection, oocytes were incubated for 4–7 days at 18°C in 1 × Ringer's solution [96 mM NaCl, 2 mM KCl, 5 mM MgCl_2_, 0.8 mM CaCl_2_, and 5 mM HEPES (pH = 7.6)] supplemented with 5% dialyzed horse serum, 50 mg/ml tetracycline, 100 mg/ml streptomycin, and 550 mg/ml sodium pyruvate.

Whole-cell currents were recorded from the injected *Xenopus* oocytes with two-electrode voltage clamp. Odorant-induced currents were recorded with an OC-725C oocyte clamp (Warner Instruments, Hamden, CT) at a holding potential of −80 mV. Odorants and DEET were dissolved in DMSO at a 1:10 ratio to make stock solutions that were diluted in 1 × Ringer's solution to the indicated concentrations (Wang et al., 2010). Between stimulations, oocytes were allowed to return to their resting potential by washing out the odorants or DEET using Ringer's solution. Recovery time was determined according to the time required for agonist-induced responses to abate and to reach pre-stimulation levels. Data acquisition and analysis were carried out with Digidata 1440A and pCLAMP 10.2 software (Axon Instruments Inc., CA).

### Olfactometer bioassay

The olfactometer bioassay was conducted by following the procedure described by Gries et al. (2015) with only minor modifications. Bioassays were conducted in dual-choice olfactometers consisting of two lateral Petri dishes (with lid) and a central dish (without lid) (3 × 9 cm inner diameter). The central dish was connected to the two lateral dishes via a plastic tube (2.5 cm long × 0.5 cm inner diameter). The dishes in this olfactometer mimic the natural still-air shelters in which bed bugs spend the day. Prior to the start of each bioassay, a disc of filter paper (9 cm diameter) was placed into each dish and a strip of filter paper (24 × 0.6 cm) inserted into the connecting tubing to provide traction for walking bed bugs. In addition, a piece of filter paper was placed into each lateral dish and covered with a piece of cardboard (2.2 × 2.2 cm) as a refuge for bioassay insects. An inverted lid of a 4-ml vial was placed on top of the corrugated cardboard shelter in the randomly assigned treatment dish of the olfactometer. All these olfactometers were placed in a small room with excellent air circulation. Before adding the stimulus or DMSO, the connected tube was sealed using a small piece of Parafilm membrane (Sigma) and a single male or female adult bed bug released into the central chamber of each of 20–60 olfactometers for each experiment at the end of the 12-h photophase. The chemical stimulus formulated in equal amounts in DMSO was then pipetted into the lid of experimental treatment, while in the control treatment only DMSO was added into the inverted lid. The bed bug in each olfactometer was then allowed to explore the central dish for 1 h of darkness, after which the Parafilm membrane was removed in the connected tubes, enabling the bed bugs to detect the odorants in either side of the dish. After the 12-h darkness period, the bed bug's position within each olfactometer was recorded. Any insect not found in a lateral chamber was recorded as a non-responder. Olfactometers were washed with unscented detergent (Beaumont Products, GA, USA), rinsed with distilled water, and dried at room temperature between each bioassay.

### Ethics approval statement

This study was carried out in accordance with the recommendations of the AVMA Guidelines for the Euthanasia of Animals: 2013 Edition. The protocol was approved by the Auburn University Animal Care and Use committee (Policy number is 2016-2987).

## Results

### DEET activated ORNs in the Dα and Dβ sensillum and multiple ORs

**Figure 1:**
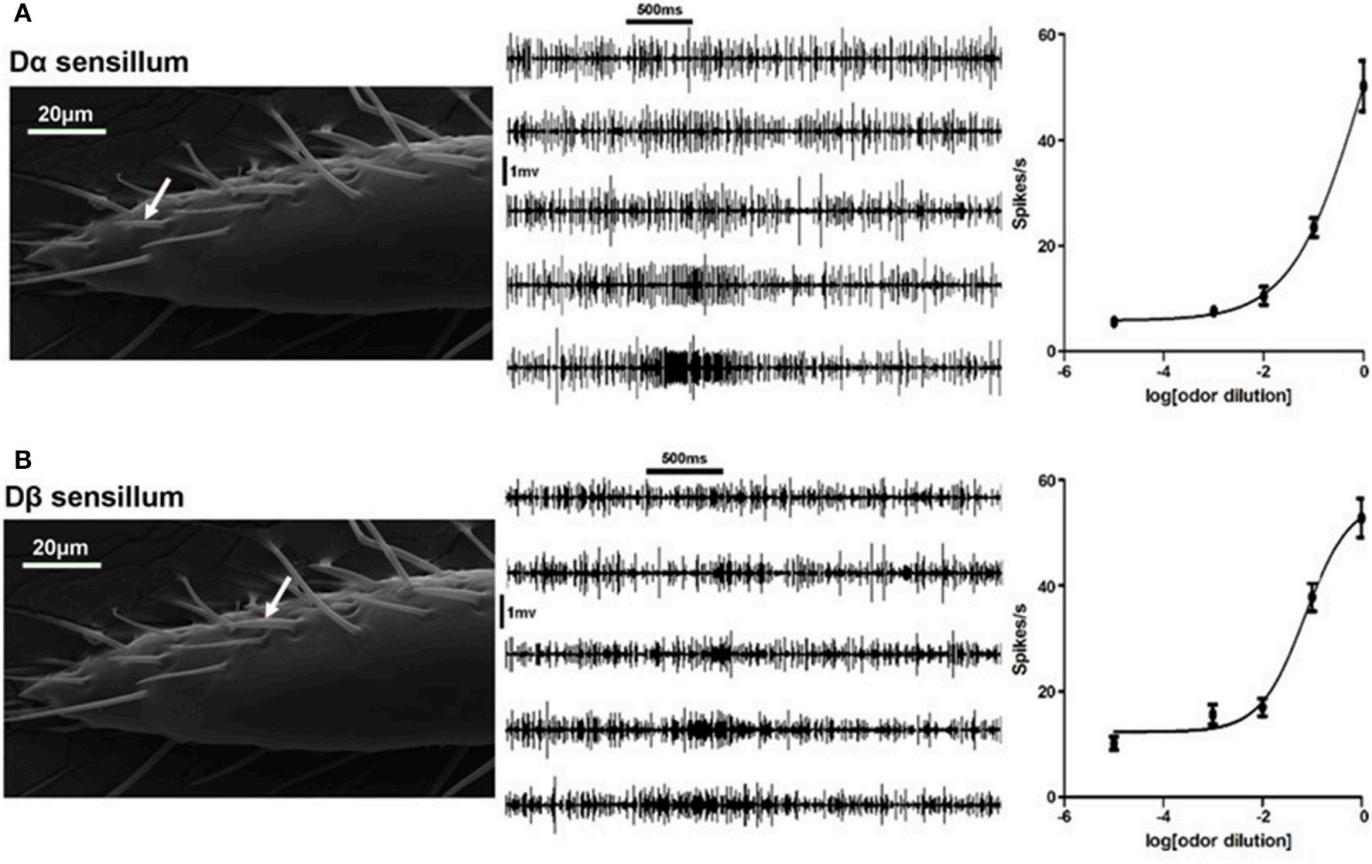
Neuronal responses of olfactory sensilla to DEET in the common bed bug. **(A)** Dose-dependent responses of the Dα sensillum (white arrow) to DEET. **(B)** Dose-dependent responses of the Dβ sensillum (white arrow) to DEET. The representative firing traces of ORNs in responses to different doses of DEET (from top to bottom: 1:10^4^−1:10^0^ v/v). The dose-response curve was fitted with the Sigmoidal dose-response model with variable slope using Graphpad Prism 5.

To test if bed bugs are able to sense DEET, we screened all types of olfactory sensillum on the bed bug antennae, including Dα, Dβ, Dγ, C, E1, and E2 sensilla, via single sensillum recording. The results showed that DEET elicited excitatory response from Dα and Dβ sensilla, particularly at high concentrations. For example, pure DEET was able to stimulate the Dα and Dβ sensilla,with the firing rate of 50 ± 4.8 and 53 ± 3.7 spikes/s, respectively (Figures 1A,B).

**Figure 2:**
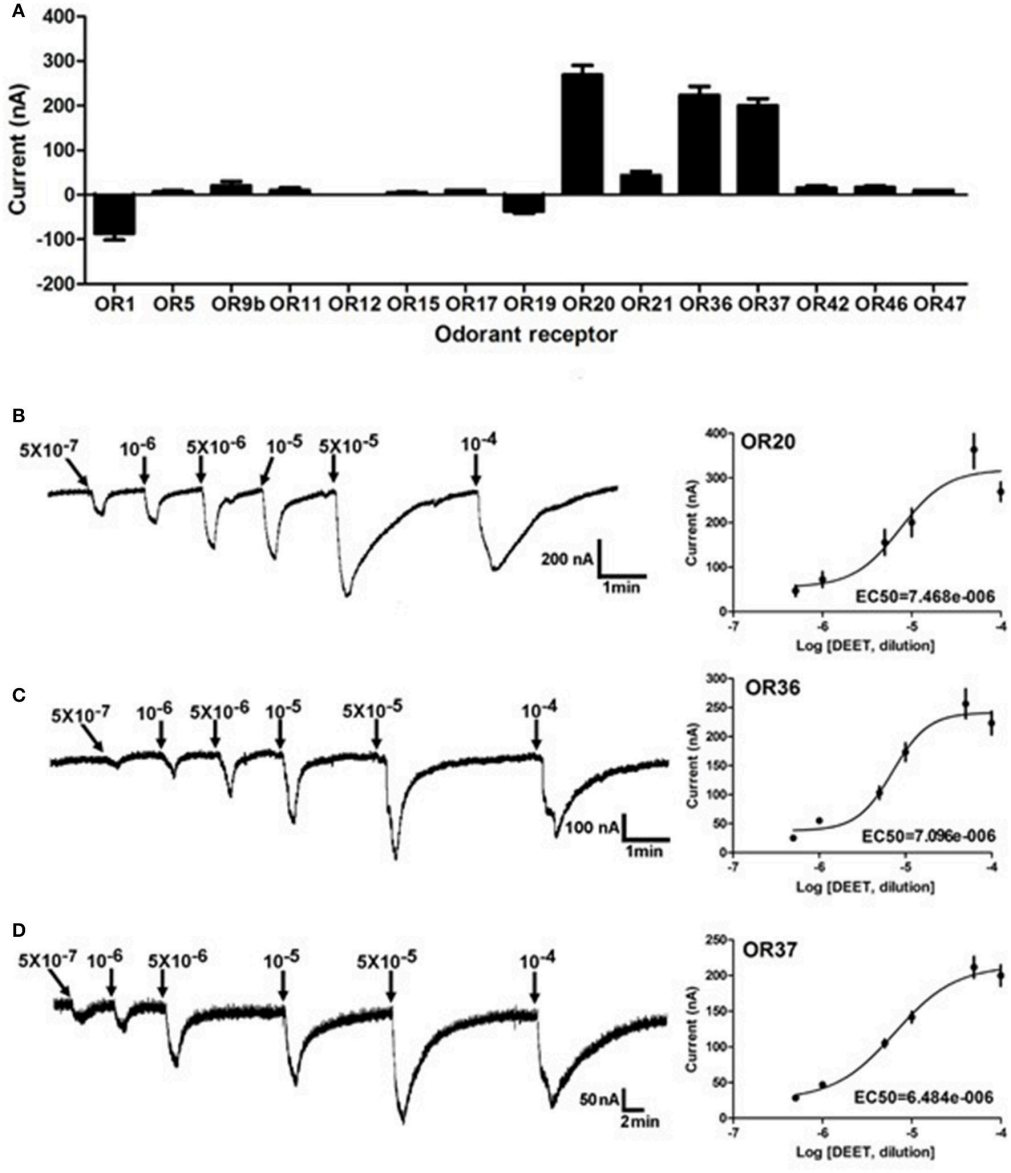
Activating effect of DEET on multiple ORs in the common bed bug. **(A)** Three out of 15 bed bug ORs showed current responses of more than 100 nA to a perfusion of DEET at a dose of 1:10^4^ v/v, *n* = 6–10; **(B)** Dose-dependent responses of OR20 to DEET from a dose of 1: 5 × 10^7^ to 1:10^4^ v/v; EC_50_ = 7.468 × 10^−6^; **(C)** Dose-dependent responses of OR36 to DEET from the dose of 1: 5 × 10^7^ to 1:10^4^ v/v; EC_50_ = 7.096 × 10^−6^; **(D)** Dose-dependent responses of OR37 to DEET from the dose of 1: 5 × 10^7^ to 1:10^4^ v/v; EC_50_ = 6.684 × 10^−6^; The values of the current responses from the OR/ORCO complex are presented as the M (mean) ± SEM. The dose-response curve was fitted with the Sigmoidal dose-response model with variable slope using Graphpad Prism 5.

Insect odorant receptors (ORs) in the dendrite membrane of ORNs are responsible for sensitizing odorants and producing the neuronal firings (action potential) (Leal, 2013). To further identify potential bed bug odorant receptor(s) that are activated by DEET, previously reported 15 bed bug ORs expressed in the *Xenopus* oocytes were challenged by DEET with two-electrode voltage clamp. The results showed that at least three of these ORs, OR20, OR36, and OR37, showed remarkable current responses (≥100 nA) to DEET (Figure 2A). The responses of these ORs to DEET also followed a dose-dependent pattern, with EC_50_ values of 7.5 × 10^−6^, 7.1 × 10^−6^, and 6.5 × 10^−6^, respectively (Figures 2B–D).

**Figure 3:**
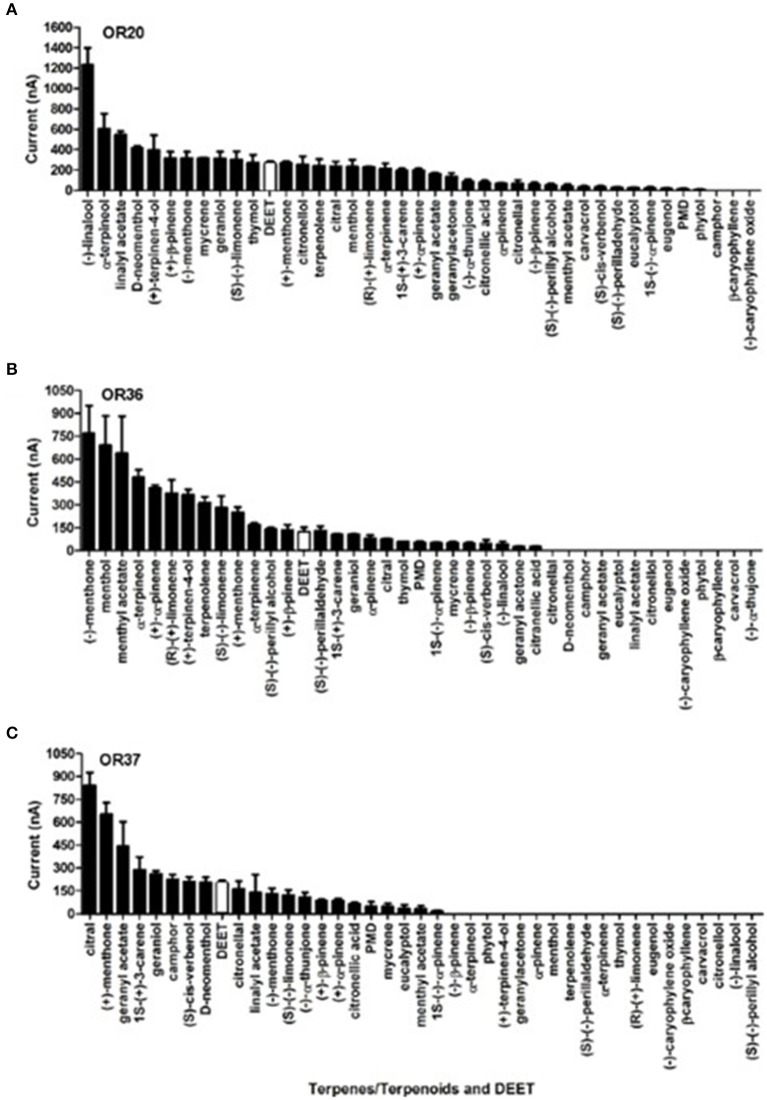
The current responses of three DEET-sensitive ORs to terpenes/terpenoids and DEET. **(A)** Responses of OR20 to terpenes/terpenoids and DEET; **(B)** Responses of OR36 to terpenes/terpenoids and DEET; **(C)** Responses of OR37 to terpenes/terpenoids and DEET. The responses to DEET are shown in white column. All the data (Mean ± SEM) from the ORs' responses to terpenes/terpenoids were retrieved from Liu et al. (2017).

**Figure 4:**
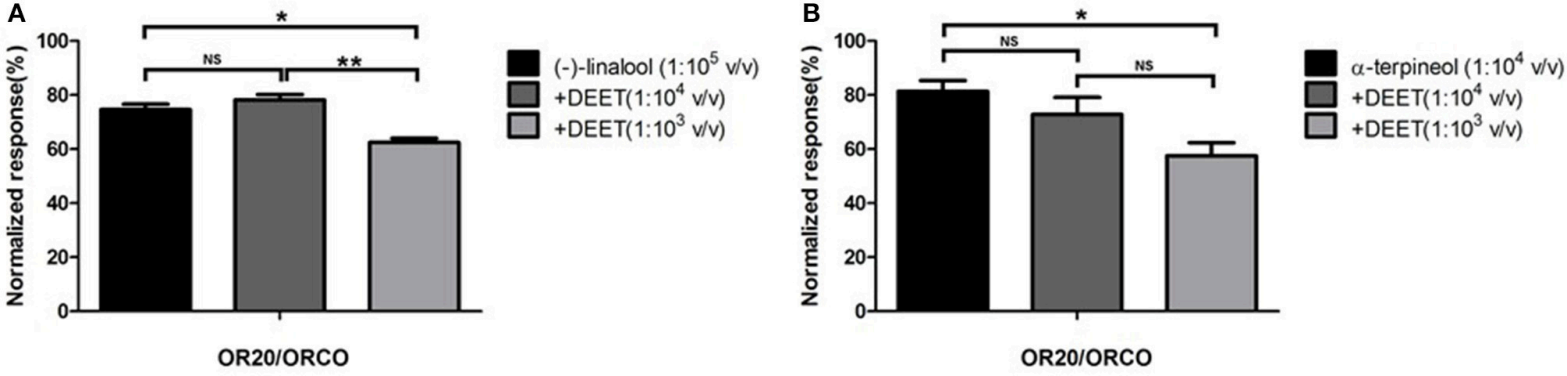
Competitive effect of DEET with terpenoids in activating OR20 of bed bugs. **(A)** DEET competed with (−)-linalool in eliciting current response from OR20/ORCO complex; **(B)** DEET competed with α-terpineol in activating OR20/ORCO. Each replicate was conducted by firstly using odorant alone to perfuse the oocyte and then continuously using the odorant or odorants/DEET mixture to perfuse the oocyte. The normalized responses of odorants or odorant/DEET mixture were defined as percentages of responses to odorant alone in the first stimulation. Significant difference was defined as ^*^ when *P* < 0.05 or ^**^ when *P* < 0.01 in the *t*-test. NS mean no significant difference.

Of particular interest is the observation that all these ORs (OR20, OR36, and OR37) that were activated by DEET were even more sensitive to certain terpenes/terpenoids than odorants from other chemical classes. Particularly, OR37 was mainly activated by terpenes/terpenoids (Liu et al., 2017). Comparatively, OR20, OR36, and OR37 showed much stronger responses to (−)-linlool, (−)-menthone and citral than to DEET (Figure 3). When we mixed DEET with (−)-linalool (Figure 4A) or α-terpineol (Figure 4B) in stimulating OR20/ORCO, the response to either (−)-linalool or α-terpineol was reduced when high dose of DEET was applied, which suggested that a competitive effect may exist between DEET and (−)-linalool/α-terpineol. As all these terpenes/terpenoids are major components of essential oils or other botanical repellents that are repulsive for blood-feeding mosquitoes, we then continued to test the behavioral response of bed bugs to DEET and terpenes/terpenoids.

### Bed bugs are behaviorally aversive to DEET and certain terpenes/terpenoids

**Figure 5:**
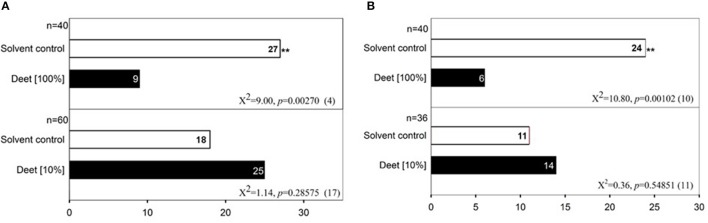
Repellency of high doses of DEET to the common bed bug. **(A)** Behavior bioassay of male bed bugs to two doses of DEET (10%, 100%); **(B)** Behavior bioassay of female bed bugs to two doses of DEET (10%, 100%). For each experiment, an asterisk indicates a significant response to DEET; χ2 test with Yates correction for continuity; ^*^P < 0.05; ^**^P < 0.01 (Siljander et al., 2008). Fifty microliters DEET solutions of different doses were applied in each test. The value of *n* indicates the replicates for the two-choice olfactometer bioassay of individual bed bugs. DMSO was used as the control solvent for each replicate. Numbers in parentheses indicate the number of bed bugs not responding to DEET.

**Figure 6:**
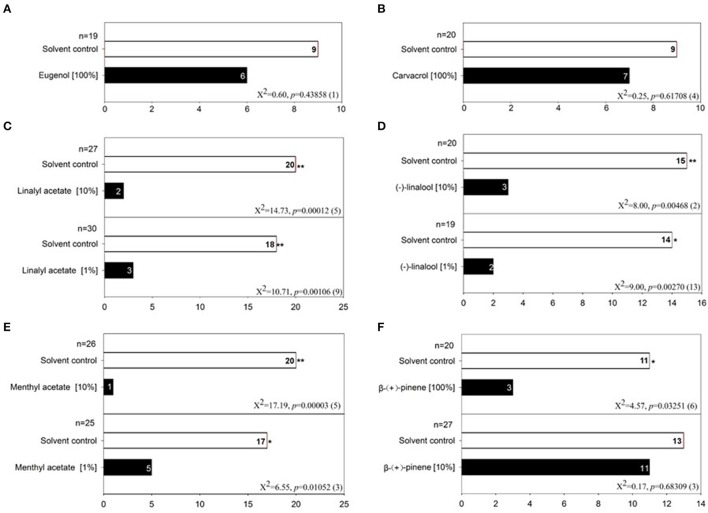
Behavior bioassay of bed bugs in response to terpenes/terpenoids. **(A)** Olfactometer bioassay of bed bugs to 100% eugenol; **(B)** Olfactometer bioassay of bed bugs to 100% carvacrol; **(C)** Olfactometer bioassay of bed bugs to two doses of linalyl acetate (1%, 10%); **(D)** Olfactometer bioassay of bed bugs to two doses of (−)-linalool (1%, 10%); **(E)** Olfactometer bioassay of bed bugs to two doses of menthyl acetate (1%, 10%); **(F)** Olfactometer bioassay of bed bugs to two doses of (+)-β-pinene (10%, 100%). For each experiment, an asterisk indicates a significant response to the treatment stimulus; χ2 test with Yates correction for continuity; ^*^P < 0.05; ^**^P < 0.01 (Siljander et al., 2008). Fifty microliters treatment stimulus of different doses was applied in each test. The value of *n* indicates the replicates for the two-choice olfactometer bioassay of individual bed bugs. DMSO was used as the control solvent for each replicate. Numbers in parentheses indicate the number of bed bugs not responding to either test stimulus.

To test the behavioral responses of bed bugs to DEET and terpenes/terpenoids, we applied a dual-choice olfactometer bioassay as described by Gries et al. (2015). With this method, we found that both male and female bed bugs were significantly repelled by pure DEET but not 10% DEET solution, which suggests that only high doses of DEET elicit an aversive response in both male (Figure 5A) and female bed bugs (Figure 5B). To test if DEET's repellency to bed bugs was due to excessive concentrations applied, we chose other two terpenoids, eugenol, and carvacrol at the concentration of ~100%, which did not activate any DEET-sensitive ORs. Unsurprisingly, both of eugenol and carvacrol showed absolutely no activity in repelling the bed bugs (Figures 6A,B), which suggested that bed bugs did sense DEET and repelled by it but not due to other physical interference. For the terpenes and terpenoids which displayed strong activation on the DEET-sensitive ORs, much more potency was observed in repelling bed bugs than that of DEET. For instance, 1% of linalyl acetate, menthyl acetate, (−)-linalool (Figures 6C–E), (+)-menthone (Figure S1A) and 5% of citral, geranyl acetate and 1S-(+)-3-carene (Figures S1B–D) already displayed very strong repellency to bed bugs. Another terpene odorant, (+)-β-pinene, which demonstrated a closing activating effect as DEET on the receptors, displayed a similar repellency to bed bugs only at high concentrations (Figure 6F).

### Interfering effect of DEET on the responses of ORNs to human odorants

**Figure 7:**
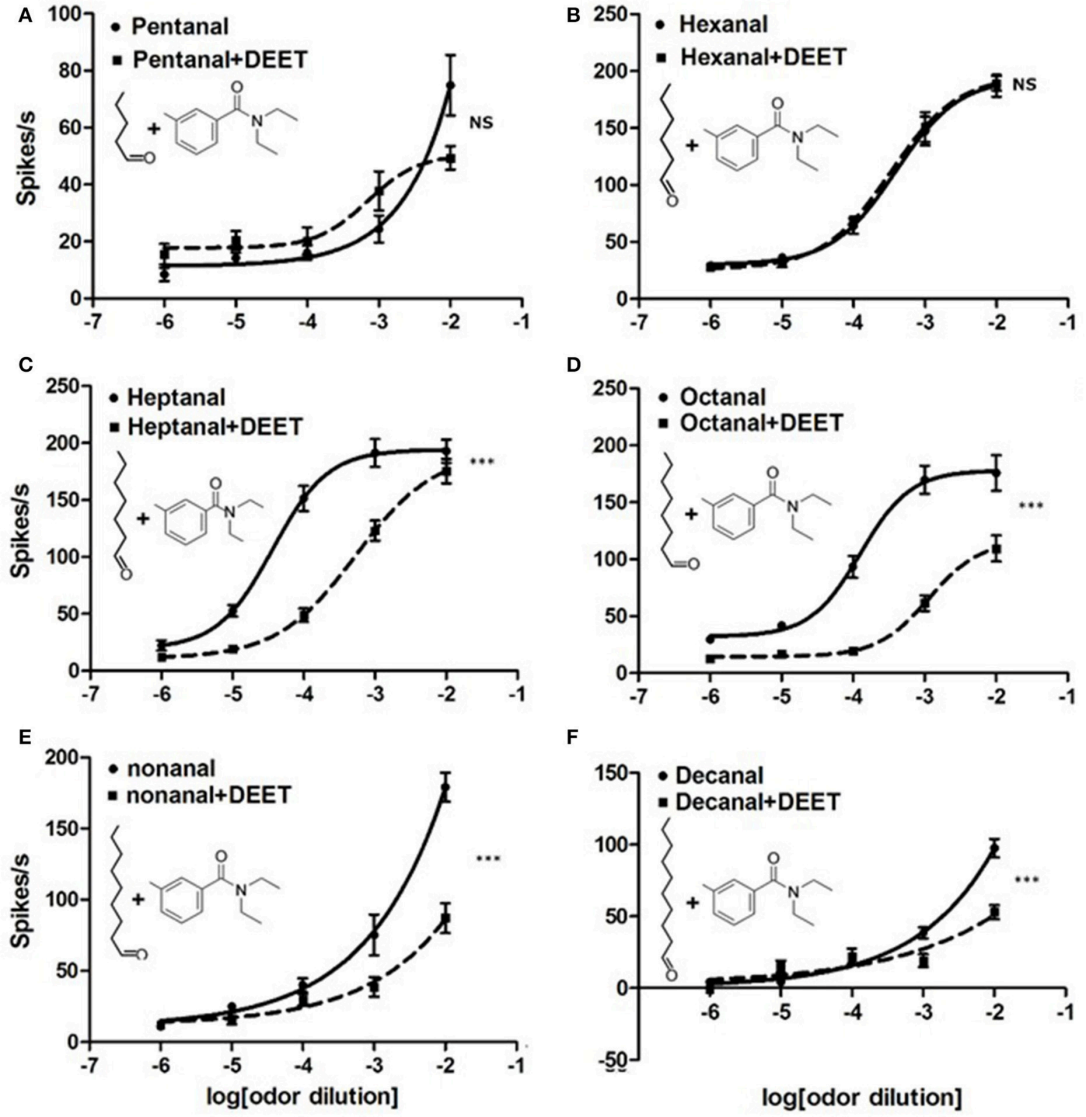
Modulation of DEET on the neuronal responses of bed bug Dγ sensilla to aldehyde odorants. **(A)** Dose-response curves of ORNs in Dγ sensilla to pentanal (1:10^2^ v/v) with (solid line) or without (dashed line) DEET; **(B)** Dose–response curves of ORNs in Dγ sensilla to hexanal (1:10^2^ v/v) with (solid line) or without (dashed line) DEET; **(C)** Dose–response curves of ORNs in Dγ sensilla to heptanal (1:10^2^ v/v) (solid line) or without (dashed line) DEET; **(D)** Dose–response curves of ORNs in Dγ sensilla to octanal (1:10^2^ v/v) (solid line) or without (dashed line) DEET; **(E)** Dose–response curves of ORNs in Dγ sensilla to nonanal (1:10^2^ v/v) (solid line) or without (dashed line) DEET; **(F)** Dose–response curves of ORNs in Dγ sensilla to decanal (1:10^2^ v/v) (solid line) or without (dashed line) DEET. (*F*-test with Bonferroni correction; mean ± SEM., *n* = 6–10; NS, no significance; ^*^P < 0.05; ^**^P < 0.01; ^***^P < 0.001). Dose-response curve was fitted with the Sigmoidal dose-response model with variable slope using Graphpad Prism 5.

Although we confirmed that DEET can activate ORNs or ORs of bed bugs directly, many previous studies on fruit fly and mosquito have also indicated that DEET might exert an interfering effect on neuronal responses to the odorants (Ditzen et al., 2008; Pellegrino et al., 2011). To investigate whether DEET displayed the same function on bed bugs' olfactory systems, we characterized the neuronal response of Dγ and C sensilla to the combination of DEET and human odorants. Human odorants from different classes were chosen based on their strong stimulation on the Dγ or C sensilla, demonstrated in our previous study (Liu and Liu, 2015). Interestingly, we found that the responses of bed bugs to human odorants were totally “scrambled” when DEET was added into the stimulation (Figure 7 and Figures S2, S3). For example, Dγ sensilla showed no significant difference in dose-dependent response to combinations of DEET and hexanal or DEET and toluene compared to hexanal or toluene alone (Figure 7B and Figure S2A). However, for some other aldehyde chemicals, including heptanal, octanal, nonanal, and decanal, a very significant blocking effect was observed in the neuronal responses to the mixtures when DEET was added, with the dose-dependent curves being much lower than those of the aldehydes alone (Figures 7C–F). It is worth noting that while the majority of the strongest blocking effects appeared at the highest concentration of human odorants, a few were presented at some medium concentration, such as heptanal at the concentration of 10^−4^, and octanal at 10^−3^(Figures 7C,D), suggesting that high concentration of heptanal and octanal can overcome the blocking effect of DEET on the ORNs, which was consistent with previous finding in *Drosophila* (Pellegrino et al., 2011). Although DEET showed an extensive blocking effect on the neuronal responses of Dγ sensilla to certain chemicals, no interfering effect was observed on the response of C sensilla to two amines tested, propylamine and butylamine (Figure S3).

**Figure 8:**
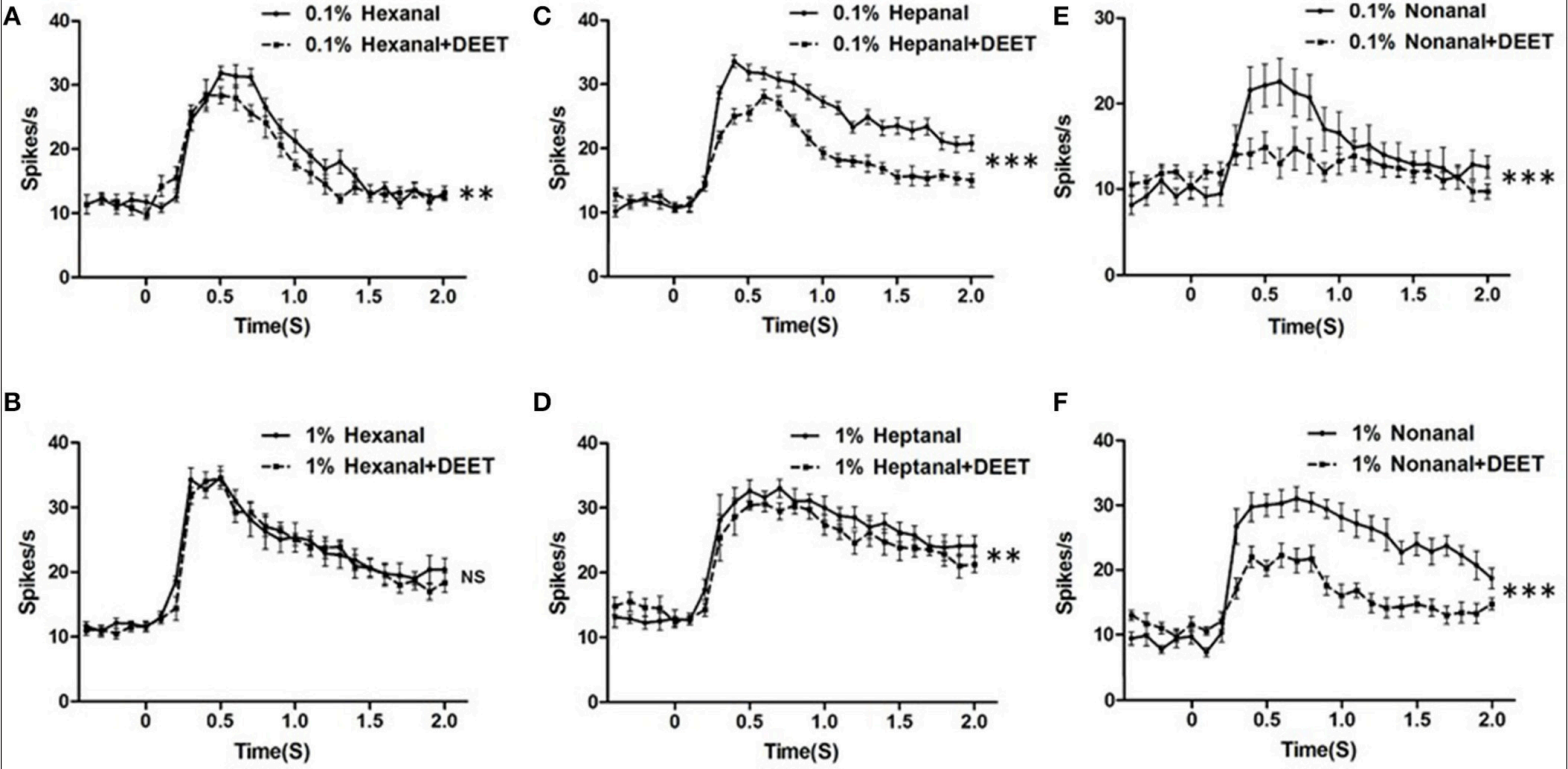
Modulation of DEET on the temporal dynamic of responses to odorants. **(A)** Temporal dynamic of responses to hexanal at a dose of 1:10^3^ v/v with (dash line) or without (solid line) DEET; **(B)** Temporal dynamic of responses to hexanal at a dose of 1:10^2^ v/v with (dash line) or without (solid line) DEET; **(C)** Temporal dynamic of responses to heptanal at a dose of 1:10^3^ v/v with (dash line) or without (solid line) DEET; **(D)** Temporal dynamic of responses to heptanal at a dose of 1:10^2^ v/v with (dash line) or without (solid line) DEET; **(E)** Temporal dynamic of responses to nonanal at a dose of 1:10^3^ v/v with (dash line) or without (solid line) DEET; **(F)** Temporal dynamic of responses to nonanal at a dose of 1:10^2^ v/v with (dash line) or without (solid line) DEET. (*F*-test with Bonferroni correction; mean ± SEM, *n* = 6–10; NS, no significance; ^*^P < 0.05; ^**^P < 0.01; ^***^P < 0.001).

The temporal dynamic of neuronal responses is also considered an important feature for odorant encoding or recognition. To test whether DEET showed any influence on the temporal characteristics of neuronal response to human odorants, we compared the temporal dynamics of the bed bugs' response to combinations of DEET and odorants with those for solely odorants. We found that DEET did change the temporal structure of the neuronal response to certain odorants and this change was both odorant-specific and dose-specific (Figure 8). For instance, DEET showed a huge modification on the temporal structure of responses to nonanal by inhibiting the peak firing from the ORNs housed in the Dγ sensillum (Figure 8C), but had no effect on the temporal structure of responses to hexanal (Figure 8A). For another odorants, DEET had a large impact on the response to heptanal, with the temporal structure shifting from more tonic to more phasic (Figure 8B). However, when the concentration of heptanal increased, DEET's effect was diminished, providing further evidence for high concentration of odorants could overcome DEET's interference on the temporal structure of neuronal responses.

### DEET inhibits the responses of odorant receptor to human odors

**Figure 9:**
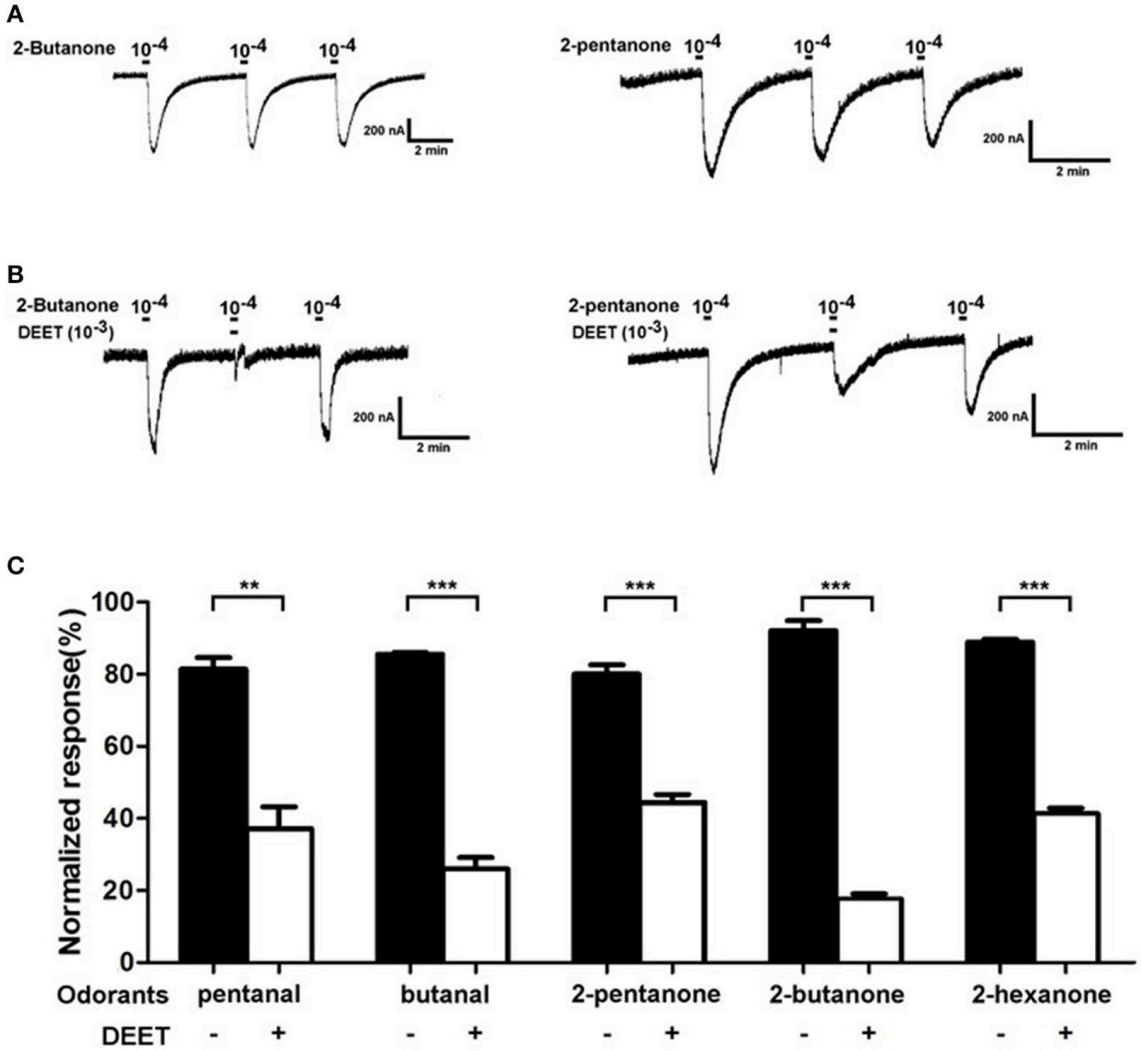
Antagonistic effect of DEET on the current responses of OR19/ORCO to odorants. **(A)** 2-butanone and 2-pentanone at a dose of 1:10^4^ v/v elicited macroscopic inward currents in oocytes expressing OR19/ORCO, respectively. During a repetitive stimulation, the agonist-evoked amplitudes are slightly desensitized; **(B)** Current response evoked by 2-butanone and 2-pentanone (at a dose of 1:10^4^ v/v), considerably inhibited by DEET (1:10^3^ v/v). **(C)** DEET significantly antagonized the current responses of OR19/ORCO to the odorants. To enable a unbiased comparison, responses evoked from a second stimulation with or without DEET are normalized with the responses evoked from the first stimulation (*t*-test with Bonferroni correction; mean ± SEM, *n* = 6; ^*^P < 0.05; ^**^P < 0.01; ^***^P < 0.001).

Previous studies on mosquitoes have proved that DEET affects the ion channel formed by the complex of odorant receptors and co-receptors, disturbing the recognition of odorant receptors to their ligands (Bohbot et al., 2011; Bohbot and Dickens, 2012). To investigate whether a similar mechanism is also involved in the repulsive effect of DEET for bed bugs, we tested the current responses of a bed bug odorant receptor (OR19) to several human-odor stimuli (pentanal, butanal, 2-butanone, 2-pentanone, 2-hexanone) both with and without DEET added into the perfusion. The results showed that all the stimuli with no DEET elicited typical strong responses in OR19/ORCO. For example, 2-butanone and 2-pentanone triggered remarkable current responses in OR19/ORCO, even though the responses decreased slightly when challenged repeatedly with high concentrations of odorants (Figure 9A). However, when DEET was added to either 2-butanone or 2-pentanone solutions, the current responses of OR19 to both stimuli decreased dramatically (Figure 9B) and this pattern was repeated in all the other human-odor stimuli tested (Figure 9C).

**Figure 10:**
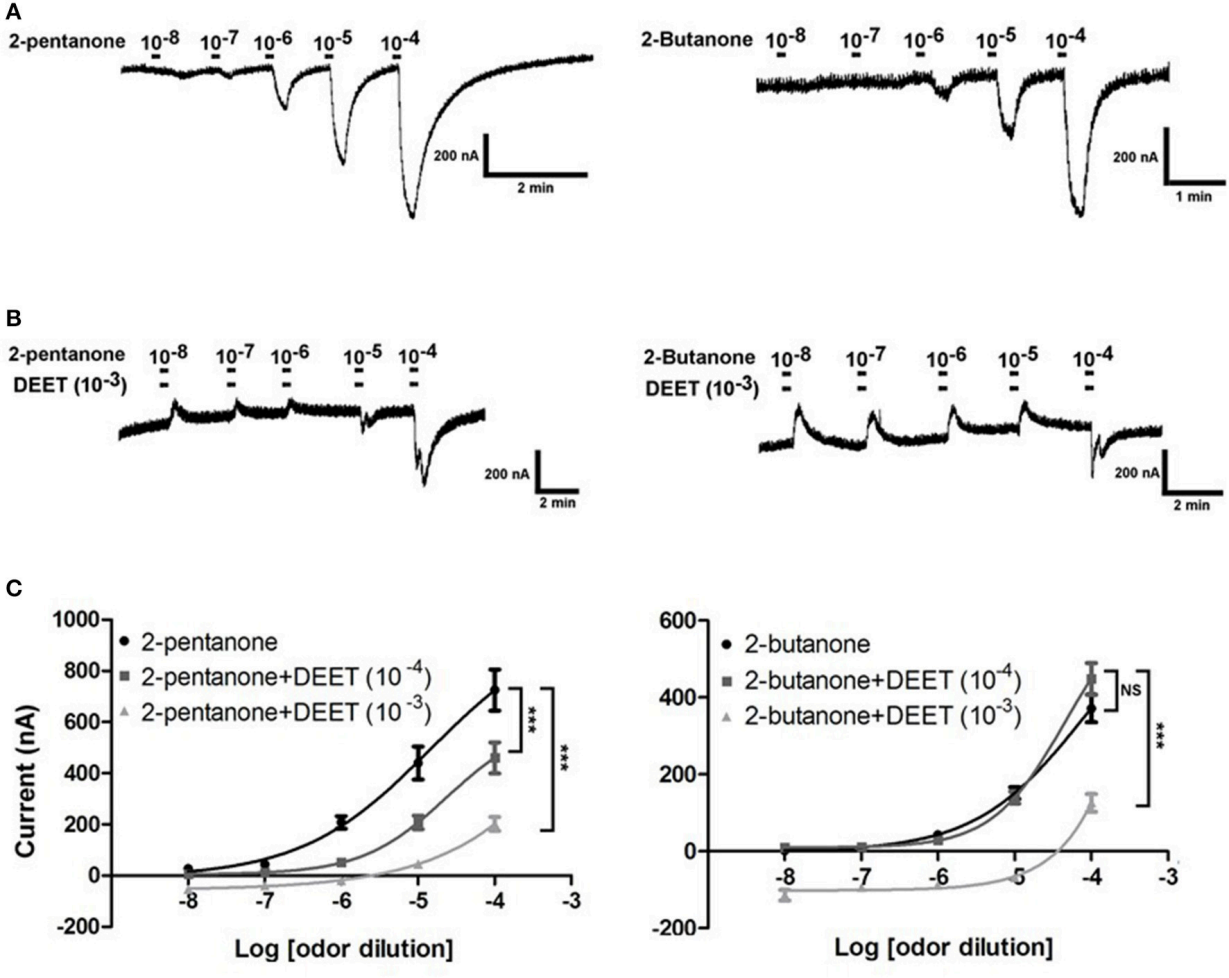
Antagonistic effect of DEET on the dose-dependent responses of OR19/ORCO to 2-butanone and 2-pentanone. **(A)** Dose-dependent responses of OR19/ORCO to 2-butanone and 2-pentanone without DEET added into the perfusion; **(B)** Dose-dependent responses of OR19/ORCO to 2-butanone and 2-pentanone with DEET (1:10^3^ v/v) added into the perfusion; **(C)** Fitted dose-response curve of OR19/ORCO to 2-pentanone and 2-pentanone with (light gray line 1:10^4^ v/v, dark gray line 1:10^3^ v/v) or without (black line) DEET (*F*-test with Bonferroni correction; mean ± SEM., *n* = 6–10; NS, no significance; ^*^P < 0.05; ^**^P < 0.01; ^***^P < 0.001). The dose-response curve was fitted with the Sigmoidal dose-response model with variable slope using Graphpad Prism 5.

The effect of DEET on the dose-dependent response of OR19/ORCO to 2-butanone and 2-pentanone was also investigated in this study. We found that DEET showed a very clear antagonistic effect to the dose-dependent responses of OR19/ORCO to both 2-butanone and 2-pentanone (Figures 10A,B). The concentrations of DEET also had a major impact on the antagonistic effect of DEET: increasing the concentration of DEET significantly enhanced the antagonistic effect. For example, the antagonistic effect of DEET on the dose-dependent response of OR19/ORCO to 2-pentanone and 2-butanone was significantly stronger at the concentration of 10^−3^ than 10^−4^ (Figure 10C).

These results clearly indicate that DEET interacts with bed bug odorant receptors to inhibit the current response of specific receptors to human odors. This antagonistic effect of DEET on one or more odorant receptors probably creates the blocking effect on the ORNs in response to human odors.

## Discussion

In this study, we characterized the neuronal responses of olfactory sensillum on the bed bug antennae to DEET and revealed that DEET activated multiple bed bug odorant receptors which also involved in detecting certain terpenes or terpenoids. Behavioral bioassays confirmed the repellency of DEET and terpenes/terpenoids on bed bugs. When we examined the constituents of some commercial insect repellents, particularly the mosquito repellents, most were labeled as containing 10-40% of DEET with a minor constituent being essential oils, largely consisting of terpenes/terpenoids. As we found in this behavioral bioassay, the thresholds for certain terpenes/terpenoids in repelling the bed bugs were much lower than that of DEET. Therefore, the minor portion of terpene/terpenoids in the commercial insect repellents may also play a significant role in protecting from mosquito biting as well as providing the fragrance, even though DEET is typically considered to play the major role in repelling the mosquitos.

In testing the neuronal responses of bed bugs to DEET, we found that although we used high doses (≥10%) of DEET in the stimulation, only mild responses were observed from both the Dα and Dβ sensilla, while very strong responses were recorded from terpenes/terpenoids in the same sensilla. This may lie in two reasons: (1) The vapor pressure of DEET is much lower than most terpene/terpenoids. Although the same concentration has been applied in stimulating the sensillum, the real amount of compounds delivered can be hugely different. (2) DEET activates the same receptor but with much smaller current response compared to terpene/terpenoids, which suggested that the binding affinity of DEET to these bed bug ORs was much lower than that of terpenes/terpenoids. Interestingly, a similar result has also been reported in the mosquito *Culex quinquefaciatus*, Syed and Leal (2008) found that specific olfactory sensilla on the antennae of *Cx. quinquefasciatus* presented much weaker response to DEET compared with several other terpenes/terpenoids.

According to the proposed “Birth-and-death” mechanism of gene evolution (Nei and Rooney, 2005; Demuth et al., 2006), insects or mammalians acquire new features or adapt to changing environment by gene duplication or expansion. However, the specificity of bed bugs (wingless, obligate blood-feeding, traumatic insemination, etc.) render bed bugs possess a evolutionarily relatively stable OR repertoire (Benoit et al., 2016) with rare gene expansion or duplication since they break with other Hemipteran insects (Benoit et al., 2016). Moreover, DEET is a synthetic odorant that has only existed for just over 70 years. Therefore, it is less likely that bed bugs or maybe other insects evolved a novel ORs that recognized DEET in such a short timeframe. Instead, we argued that DEET was accidentally introduced into the chemical ecology of bed bugs or other insects and activated the same receptors of certain terpenes/terpenoids, which presents very unpleasant odors to the insects. In addition, terpenes/terpenoids are found to be broadly detected by the ORs or ORNs of various insect species (Hallem and Carlson, 2006; Qiu et al., 2006; Ghaninia et al., 2007; Hill et al., 2009; Carey et al., 2010; Wang et al., 2010; Liu et al., 2013, 2014), some of these ORs may simultaneously possess the capacity of sensing DEET, which provides a possible explanation for why DEET exhibits such a broad spectrum in repelling different insect species. To test if DEET shares the same neural circuit as certain terpenes/terpenoids, further work on the calcium imaging shall make great contribution.

In addition to the activating effect of DEET on the ORNs or odorant receptors, we also found that DEET demonstrated an interference effect on the process of human odor sensation for bed bugs. It is interesting to note that DEET scrambled the odor coding process of ORNs in the Dγ sensillum to most human odors, while no significant influence was presented on the C sensillum. Morphologically, Dγ sensilla are close to the short blunted trichoid sensilla, with ORNs expressing ORs, while C sensilla resemble the coeloconic sensilla (Levinson et al., 1974), with ORNs expressing ionotropical receptors (IRs) in *D. melanogaster* (32). ORs have been proven to detect a number of alcohols, aldehydes, esters and aromatics (Hallem and Carlson, 2006; Carey et al., 2010; Wang et al., 2010), while IRs are known to be responsible for detecting many polar molecules, such as acids and amines (Abuin et al., 2011; Joseph and Carlson, 2015; McBride, 2016). Previous studies have also shown that DEET interferes with the function of mosquito ORs in the recognition of 1-octen-3-ol, a human odor (Bohbot and Dickens, 2012). The findings in this study suggested that DEET blocked the responses of ORNs to odorants probably due to the interaction with ORs in ORNs. With little has been done regarding the interaction between IRs and DEET, our work indicated that DEET had no effect on the ORNs' response to the amines tested, suggesting that DEET may have no interfering effect on the function of IRs in response to certain odorants.

As we found in this study, DEET clearly interfered with the functioning of the OR/ORCO complex and changed the binding affinity of chemical ligands to the OR/ORCO complex. However, as yet there is no direct proof to reveal which part of this complex, OR or ORCO, that DEET targets. Since DEET has been shown repulsive effects to a wide spectrum of insects, Ditzen et al. (2008) proposed that DEET may act on the ORCO, which is highly conserved among different insects. However, Tsitoura et al. (2015) reported no inhibition of ORCO even at 10 mM DEET in a study using the lepidopteran insect cell system to express the *Ae. aegypti* ORCO, suggesting that DEET has no influence on the function of ORCO. Moreover, in our study and also the work from Xu et al. (2014), OR was actually found to be activated by DEET. If DEET inhibits the function of ORCO, it would be impossible to observe an inward current response of these *in vitro*-expressed ORs to DEET. Therefore, we consider that DEET is more likely to work on the ORs rather than ORCO when interfering with the function of the OR/ORCO complex in the insect olfactory system.

## Author contributions

Conceived and designed the study: NL. Performed the experiments: FL and XX. Prepare the materials: NL. Wrote the paper: NL and FL. All authors reviewed the manuscript.

### Conflict of interest statement

The authors declare that the research was conducted in the absence of any commercial or financial relationships that could be construed as a potential conflict of interest.
