# Supplementary material for: Molecular Basis of N,N-Diethyl-3-Methylbenzamide (DEET) in Repelling the Common Bed Bug, Cimex lectularius
Source: Front Physiol. 2017 Jun 20;8:418. doi: 10.3389/fphys.2017.00418 (PMC5476731; doi:10.3389/fphys.2017.00418)
Supplement: Supplementary file 1 [file Image1.pdf]

Dual Roles of DEET in Repelling the Common Bed Bug, *Cimex lectularius*

Feng Liu, Xiaoming Xia, Nannan Liu[§]

Supplement Information

Figure legend

Figure S1. Behavior bioassay of bed bugs in response to terpenes/terpenoids**.** A) Olfactometer bioassay of bed bugs to three doses of (+)-menthone (1%, 5%, 10%); B) Olfactometer bioassay of bed bugs to two doses of citral (1%, 5%, 10%); C) Olfactometer bioassay of bed bugs to two doses of 1S-(+)-3-carene (1%, 5%, 10%); D) Olfactometer bioassay of male bed bugs to three doses of geranyl acetate (1%, 5%, 10%). For each experiment, an asterisk indicates a significant response to the treatment stimulus; $\chi 2$ test with Yates correction for continuity; *$P<0.05$; **$P<0.01$ (Siljander et al., 2008). 50 µl treatment stimulus of different doses was applied in each test. The value of n indicates the replicates for the two-choice olfactometer bioassay of individual bed bugs. DMSO was used as the control solvent for each replicate. Numbers in parentheses indicate the number of bed bugs not responding to either test stimulus.

Figure S2. **Modulation of DEET on the neuronal responses of bed bug Dγ sensilla to aromatic/aliphatic odorants.** A) Dose–response curves of ORNs in Dγ sensilla to toluene ($1:10^2$ v/v) with (solid line) or without (dashed line) DEET; B) Dose–response curves of ORNs in Dγ sensilla to xylene ($1:10^2$ v/v) with (solid line) or without (dashed line) DEET; C) Dose–

response curves of ORNs in Dγ sensilla to styrene (1:10^2 v/v) (solid line) or without (dashed line) DEET; D) Dose–response curves of ORNs in Dγ sensilla to propylbenzene (1:10^2 v/v) (solid line) or without (dashed line) DEET; E) Dose–response curves of ORNs in Dγ sensilla to ethylbenzene (1:10^2 v/v) (solid line) or without (dashed line) DEET; F) Dose–response curves of ORNs in Dγ sensilla to 2,4-dimethylhexane (1:10^2 v/v) (solid line) or without (dashed line) DEET. (F-test with Bonferroni correction; mean±SEM., n=6–10; NS, no significance; *P<0.05; **P<0.01; ***P<0.001). The dose-response curve was fitted with the Sigmoidal dose-response model with variable slope using Graphpad Prism 5.

Figure S3. No impact of DEET on the neuronal responses of bed bug Dγ sensilla to amine odorants. A) Dose–response curves of ORNs in C sensilla to propylamine (1:10^2 v/v) with (solid line) or without (dashed line) DEET; B) Dose–response curves of ORNs in C sensilla to butylamine (1:10^2 v/v) with (solid line) or without (dashed line) DEET. F-test with Bonferroni correction; mean±SEM., n=6–10; NS, no significance; *P<0.05; **P<0.01; ***P<0.001). The dose-response curve was fitted with the Sigmoidal dose-response model with variable slope using Graphpad Prism 5.

Figure S1

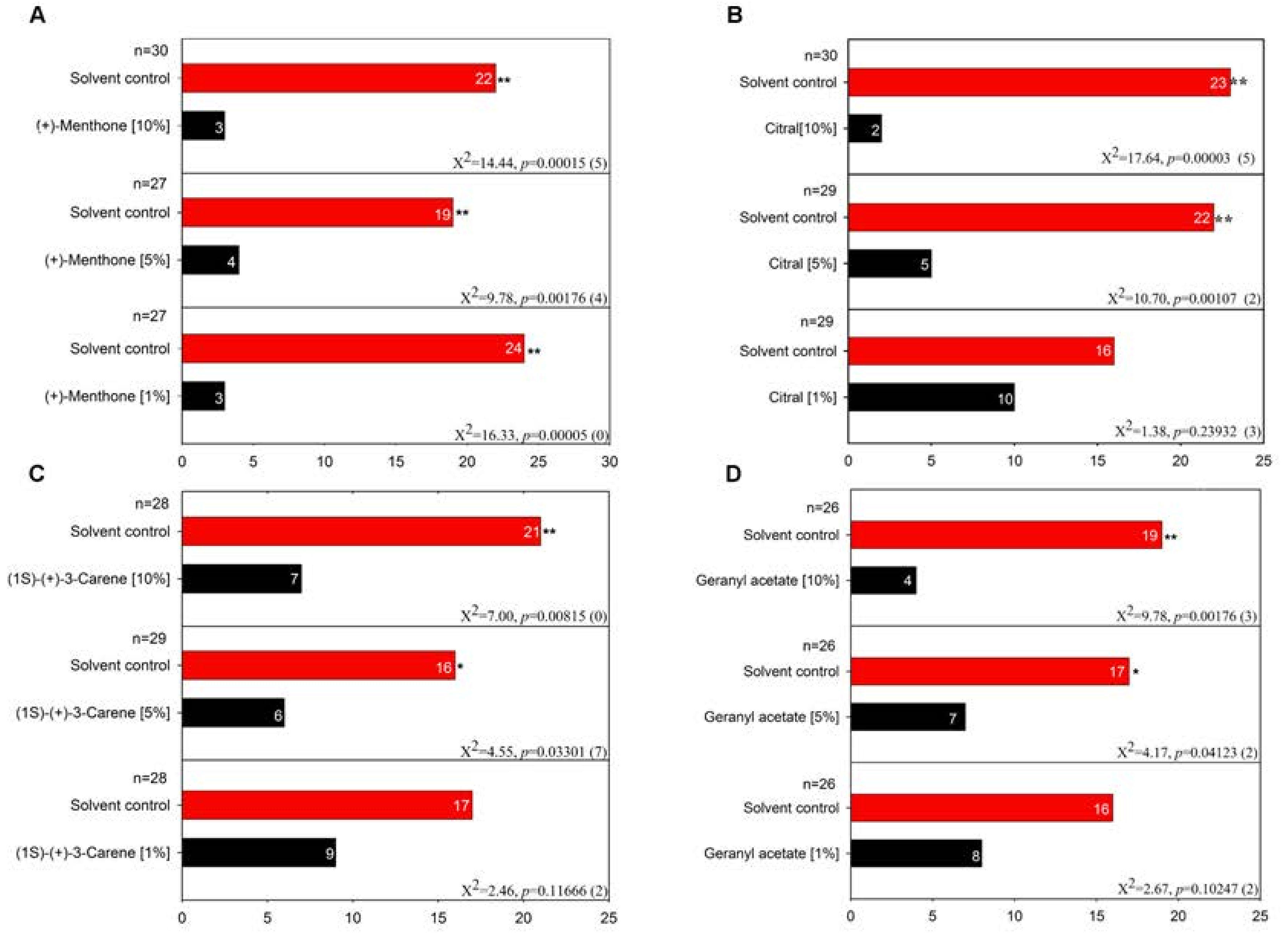
A
n=30
Solvent control
22 **
(+)-Menthone [10%]
3
X2=14.44, p=0.00015 (5)
n=27
Solvent control
19 **
(+)-Menthone [5%]
4
X2=9.78, p=0.00176 (4)
n=27
Solvent control
24 **
(+)-Menthone [1%]
3
X2=16.33, p=0.00005 (0)
0 5 10 15 20 25 30
B
n=30
Solvent control
23 **
Citral[10%]
2
X2=17.64, p=0.00003 (5)
n=29
Solvent control
22 **
Citral [5%]
5
X2=10.70, p=0.00107 (2)
n=29
Solvent control
16
Citral [1%]
10
X2=1.38, p=0.23932 (3)
0 5 10 15 20 25
C
n=28
Solvent control
21 **
(1S)-(+)-3-Carene [10%]
7
X2=7.00, p=0.00815 (0)
n=29
Solvent control
16 *
(1S)-(+)-3-Carene [5%]
6
X2=4.55, p=0.03301 (7)
n=28
Solvent control
17
(1S)-(+)-3-Carene [1%]
9
X2=2.46, p=0.11666 (2)
0 5 10 15 20 25
D
n=26
Solvent control
19 **
Geranyl acetate [10%]
4
X2=9.78, p=0.00176 (3)
n=26
Solvent control
17 *
Geranyl acetate [5%]
7
X2=4.17, p=0.04123 (2)
n=26
Solvent control
16
Geranyl acetate [1%]
8
X2=2.67, p=0.10247 (2)
0 5 10 15 20 25
