# Supplementary figures and images for: Molecular Basis of N,N-Diethyl-3-Methylbenzamide (DEET) in Repelling the Common Bed Bug, Cimex lectularius
Source: Front Physiol. 2017 Jun 20;8:418. doi: 10.3389/fphys.2017.00418 (PMC5476731; doi:10.3389/fphys.2017.00418)

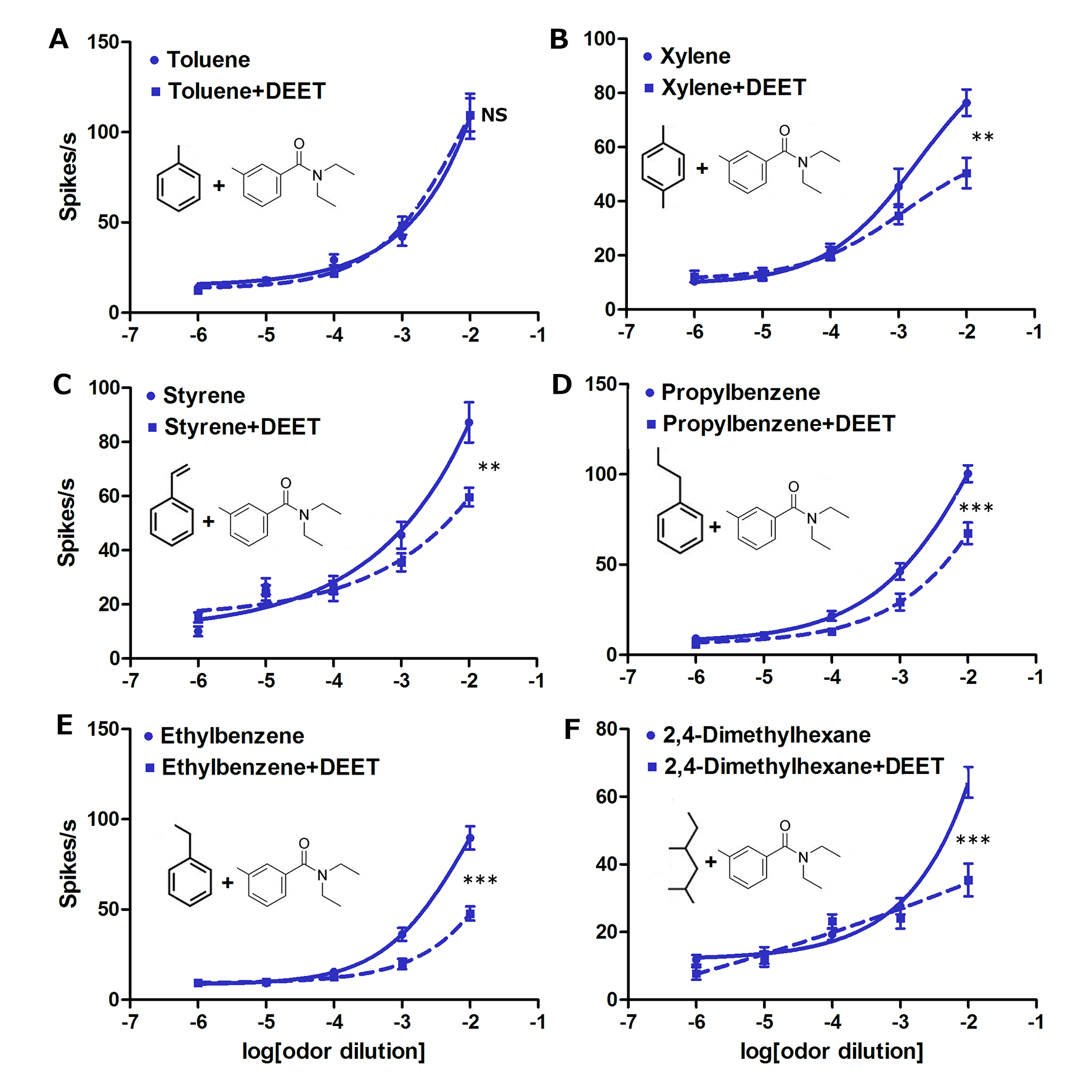

Supplement: Supplementary file 2 [file Image2.tif]

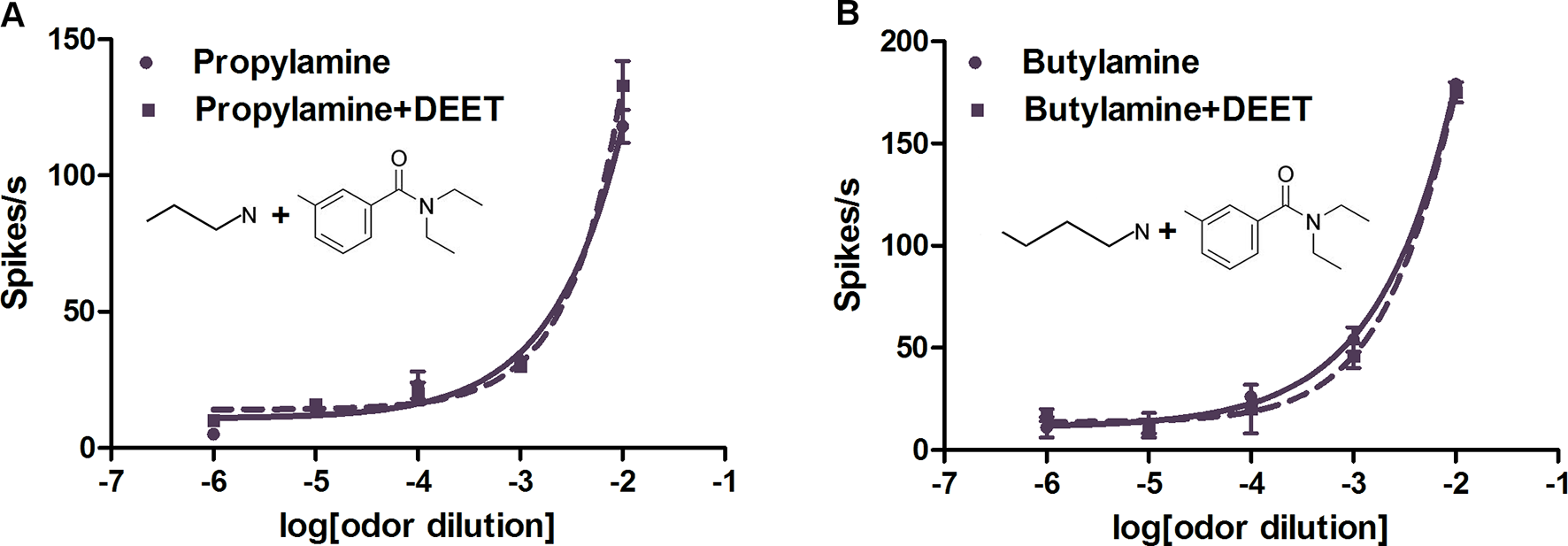

Supplement: Supplementary file 3 [file Image3.tif]
